# Implementation of Integrated Telemedicine Services in Resource‐Limited Somalia: A Case Study of Baano Healthcare Technology

**DOI:** 10.1155/ijta/3667956

**Published:** 2026-04-15

**Authors:** Najib Isse Dirie, Mohamed Mustaf Ahmed, Ahmed Adam Mohamed

**Affiliations:** ^1^ Department of Urology, Dr. Sumait Hospital, Faculty of Medicine and Health Sciences, SIMAD University, Mogadishu, Somalia, simad.edu.so; ^2^ Department of Research, Mogadishu Institute of Health, Mogadishu, Somalia; ^3^ Faculty of Medicine and Health Sciences, SIMAD University, Mogadishu, Somalia, simad.edu.so; ^4^ Health Systems Strengthening, Federal Ministry of Health, Mogadishu, Somalia, fmoh.gov.sd

**Keywords:** digital health, healthcare access, mHealth, resource-limited settings, Somalia, telemedicine

## Abstract

**Background:**

Somalia′s healthcare system faces significant challenges due to limited infrastructure and physician density (2.5 per 10,000 population). Telemedicine is a promising solution, particularly given the country′s mobile phone penetration rate of 54%. This study evaluated the implementation and impact of Baano Healthcare Technology′s integrated telemedicine platform in Somalia, while also situating its utilization within the broader disparities in healthcare access, internet coverage, and socioeconomic context.

**Methods:**

A descriptive quantitative analysis of operational data from Baano Healthcare Technology telemedicine services was conducted between July and October 2024. These services were delivered through an integrated digital platform that linked video consultations, hospital bookings, and interactive voice response self‐management services within a single telemedicine system. Data were collected through three primary channels: digital consultation, hospital bookings, and IVR self‐management services. Statistical analysis was performed using R programming software Version 4.4.0, and descriptive statistics and frequency distributions of service utilization patterns were calculated. Demographic data for digital users included age, sex, and residence, whereas IVR records lacked user‐level metadata, which limited stratification.

**Results:**

This study analyzed 610 users of video consultation and hospital booking services, along with 157,660 interactive voice response system interactions. The analysis revealed that 63.44% of users were aged 1–30 years, with a balanced sex distribution (50.82% male and 49.18% female). Hospital bookings constituted 73.61% of the services, whereas online consultations accounted for 26.39%. The Banadir region accounted for 80.49% of all users in the study. Dental services were the most requested specialty (42.98%), reflecting the scarcity of licensed dentists outside Mogadishu and the platform′s role in facilitating access to rare specialties in the region. The IVR system was substantially used for chronic condition management (47%), with diabetes management being the most frequently accessed topic (23%).

**Conclusion:**

The implementation of integrated telemedicine services in Somalia demonstrates promising potential for expanding healthcare access, particularly in urban areas. However, its reach remains constrained by geographic and digital divides, with rural areas facing compounded barriers of poverty, provider scarcity, and low internet use. The platform′s success in urban areas provides a model for expansion, although addressing infrastructure limitations and regulatory frameworks remains important.

## 1. Introduction

Telemedicine, a rapidly evolving field in healthcare delivery, has emerged as a transformative solution for addressing the challenges of accessibility, quality, and efficiency in healthcare systems worldwide [1, 2]. Defined as the provision of healthcare services and information via remote technologies, telehealth encompasses a wide range of applications including telediagnosis, telemonitoring, teletriage, teleintervention, and remote patient monitoring [3]. In recent years, the telemedicine sector has demonstrated remarkable expansion, with its global market value reaching $113.04 billion by 2025. This growth trajectory is expected to continue, with projections indicating a value of $441.35 billion by 2034, growing at a compound annual growth rate of 17.27% [4]. The advent of advanced information and communication technologies has been instrumental in driving this shift towards telemedicine, offering unprecedented opportunities to expand healthcare coverage and improve patient outcomes [5]. By complementing traditional in‐person visits, particularly for follow‐ups, routine check‐ins, and minor issues, telehealth ensures that patients seek in‐person care only when necessary, thereby optimizing healthcare resource utilization [6]. The global expansion of telehealth has been particularly pronounced in rural and remote settings, where access to healthcare services has historically been limited [5, 6]. Successful telemedicine initiatives across various countries have had a significant impact on healthcare accessibility, cost‐effectiveness, and patient satisfaction. For instance, in Australia, telemedicine services have been effectively employed to bridge the healthcare gap between urban and rural areas, improving access to specialized care for remote populations [7]. Similarly, African countries, such as Kenya, Tanzania, and Uganda, have leveraged telemedicine to overcome geographical barriers and resource constraints, highlighting the potential of these technologies in resource‐limited settings [7, 8].

In Somalia, the healthcare system faces numerous challenges that significantly impede the delivery of quality care to the population. The healthcare landscape in Somalia faces significant challenges due to decades of conflict, limited infrastructure, and resource constraints, with only 2.5 physicians per 10,000 people, making it one of the lowest physician densities globally [9]. The emergence of telemedicine and digital health solutions presents a promising avenue for addressing these barriers, particularly in regions where traditional healthcare delivery faces substantial obstacles. This digital transformation in healthcare delivery has relevance for developing nations, such as Somalia, where mobile phone penetration exceeds 54% of the population, creating a potential platform for healthcare service delivery [10]. Somalia′s formal healthcare infrastructure is heavily skewed towards basic primary care outlets and urban centers [11]. The 2022–2023 Harmonized Health Facility Assessment mapped 641 functional facilities nationwide: 3 national referral hospitals, 26 regional hospitals, and 35 district hospitals; however, 69% of all outlets are health centers or primary health units [11, 12]. Facility density averages 1.69 facilities per 10,000 population (0.93 private and 0.76 public) [13]; however, more than half of rural districts in Galmudug and Jubaland report < 0.5 facilities per 10,000, and fewer than one‐third host any district‐level hospital [11, 13, 14]. In contrast, over 60% of secondary and tertiary hospitals are concentrated in the Banadir region, reflecting decades of conflict‐driven urban migration and private‐sector growth [14]. This stark maldistribution leaves large swathes of the country reliant on underresourced primary units and underscores the potential of digital platforms to bridge specialist care gaps.

The Somali healthcare system′s current challenges, including limited specialist access, geographical barriers, and security concerns, have created an urgent need for innovative healthcare delivery solutions [15, 16]. Telemedicine platforms that integrate voice, video, and text‐based services have emerged as viable alternatives to traditional healthcare delivery methods, especially in urban and remote areas where physical healthcare infrastructure is limited [15]. Digital health initiatives such as Baano Healthcare Technology represent innovative responses to these challenges, offering an integrated telemedicine platform that combines video consultations, voice‐based health information services, SMS health updates, and hospital booking systems [17]. Several other digital health actors are now operating in Somalia, signaling an emerging but still fragmented ecosystem. SomDoctor, a Mogadishu‐based telemedicine center, provides virtual doctor consultations, diagnoses and follow‐up care, issues digital prescriptions, and runs a mobile pharmacy service that delivers medicines to patients′ homes via its iOS/Android app and web portal [16, 18]. Hello Caafi!, launched in 2021, offers remote medical and mental health consultations through a national toll‐free three‐digit phone line.

The service has already reached patients in more than 70 rural communities and positions itself as Somalia′s first dedicated telehealth platform, with a strong focus on women and hard‐to‐reach pastoral areas of the country [16, 19]. International partners are also supporting facility‐based telemedicine: in January 2021, the International Organization for Migration (IOM), together with Somalia′s Ministry of Health, began equipping several hospitals and health clinics with video‐conference screens, cameras, PC, other conferencing hardware, and 50 iPads, so that frontline clinicians can consult medical experts abroad and elsewhere in Somalia. Although each initiative pursues different use cases and payment models, together they point to growing consumer acceptance of remote care and underscore the need for coherent coordination and regulation as Somalia′s digital health sector scales. This comprehensive approach addresses multiple barriers to healthcare access, including digital literacy variations, internet accessibility constraints, and the need for both emergency and routine medical consultations. Despite the growing adoption of telemedicine services in Somalia, there is a notable gap in the empirical research examining the implementation and impact of such integrated digital health platforms. This study is aimed at assessing the implementation and impact of Baano Healthcare Technology′s integrated telemedicine services in Somalia, focusing on service utilization patterns, demographic reach, and effectiveness of various digital health delivery modalities. Understanding these aspects is crucial for informing future digital health initiatives and policy frameworks in resource‐constrained settings.

## 2. Methods

### 2.1. Study Design and Setting

This study employed a descriptive quantitative analysis design to examine operational data from Baano Healthcare Technology′s telemedicine services in Somalia. Baano Healthcare Technology, established in 2024 and headquartered in Mogadishu, is a private telehealth company operating on a fee‐for‐service model through a mobile application (iOS/Android) and a web portal. The platform provides a range of services, including live video consultations, hospital bookings, medicine delivery (e‐pharmacy), home sample collection with online laboratory results, a toll‐free helpline, and agent‐assisted bookings. These features are prominently displayed on the company website [17]. The company functions entirely as a private entity, distinct from nongovernmental organizations and government programs. Since its inception, usage analytics, as summarized in the present manuscript, have indicated consistent growth, with over 400 clinicians, primarily based in Banadir but also representing all federal states, registered to provide online and in‐person care. These initial usage records constitute the foundation of the utilization analysis presented in this study [17]. The study covered a 4‐month period from July 2024 to October 2024, encompassing service implementation and utilization patterns across multiple regions in Somalia.

### 2.2. Data Sources and Study Variables

This study drew from operational records maintained by Baano Healthcare Technology′s integrated digital health platform, extracting information from three principal service channels: a digital platform for managing video consultations, an interface for processing hospital appointments, and an automated system for tracking interactive voice response (IVR) self‐management service interactions. At the time of data extraction, Baano′s registration form required only age, self‐reported sex, and state of residence to minimize sign‐up friction; fields for occupation, education, income, and comorbidity were not yet implemented and are therefore absent from the study dataset. The IVR system operates through unstructured supplementary service data (USSD) technology, enabling users to access voice recordings without requiring internet connectivity or smartphones. The IVR provided by the mobile network operator contained only timestamps, call duration, and selected menu topic; age, gender, and other subscriber details were not captured at the source and were therefore unavailable for analysis. The demographic analysis included 610 users who accessed video consultation and hospital booking services during the specified period, whereas the IVR system recorded 157,660 interactions.

### 2.3. Data Analysis

The analysis incorporated demographic variables and service utilization metrics including sex distribution, geographic location, age group distribution, video consultation frequencies, hospital booking patterns, and IVR interaction volumes. Statistical analysis was conducted using R programming software Version 4.4.0, calculating descriptive statistics such as means, medians, and standard deviations for continuous variables, along with frequency distributions for categorical variables. The analytical approach focused on characterizing service utilization patterns across different modalities and demographic groups, where data were available.

### 2.4. Quality Control and Data Management

Data quality assurance was maintained through rigorous control measures throughout the collection and analysis process. To protect user privacy, all personal identifiers were removed during data extraction. All data were handled in accordance with data protection regulations and institutional policies.

## 3. Results

### 3.1. Demographic Characteristics of Service Users

**Figure 1:**
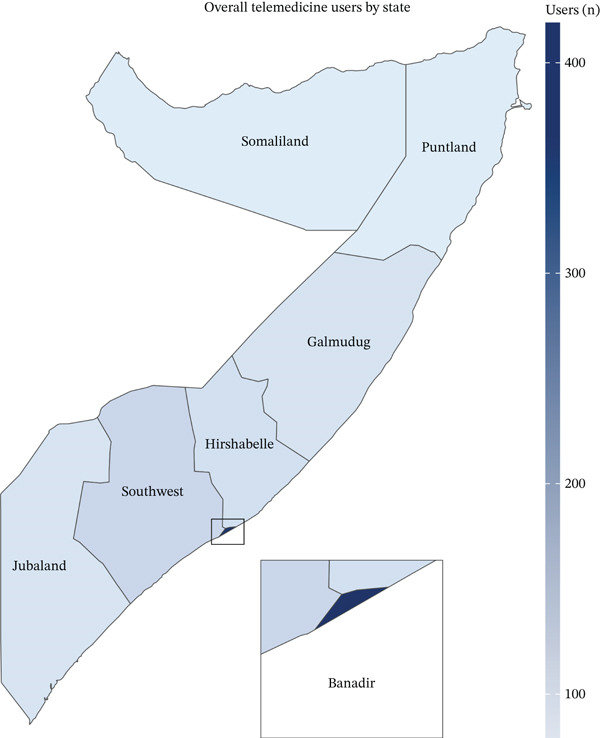
Geographic distribution of telemedicine service users across Somali states.

Demographic analysis encompassed 610 telemedicine service users, revealing distinct patterns in age distribution, sex representation, and geographical utilization. The age distribution demonstrated a predominantly young user base, with 63.44% (*n* = 387) of users falling within the 1–30 years age bracket, whereas 17.7% (*n* = 108) were between 31 and 45 years, and 18.85% (*n* = 115) were 46 years old or older (Table 1). The mean age of the users was 30.88 years (SD = 18.25), indicating a relatively young user population. The sex distribution showed an almost equal representation, with males comprising 50.82% (*n* = 310) and females comprising 49.18% (*n* = 300) of the total user base. The geographical distribution revealed a significant concentration in the Banadir state (Figure 1), which accounted for 80.49% (*n* = 491) of all users, followed by the Southwest state at 8.2% (*n* = 50) and Hirshabelle at 4.75% (*n* = 29).

**Table 1:** Demographic characteristics of telemedicine service users (*n* = 610).

Variable	Frequency	Percentage
Age (years)		
1–30	387	63.44%
31–45	108	17.7%
46+	115	18.85%
Mean ± SD	30.88 ± 18.25	
Sex		
Male	310	50.82%
Female	300	49.18%
State		
Banadir	491	80.49%
Southwest	50	8.2%
Hirshabelle	29	4.75%
Galmudug	20	3.28%
Jubaland	17	2.79%
Puntland	2	0.33%
Somaliland	1	0.16%

### 3.2. Service Utilization Patterns

The analysis of service utilization patterns demonstrated that online bookings constituted most telemedicine interactions, accounting for 73.61% (*n* = 449) of the total services, whereas online consultations represented 26.39% (*n* = 161) of all interactions (Table 2).

**Table 2:** Service utilization patterns across different telemedicine modalities (n = 610).

Category	Frequency	Percentage
Online bookings	449	73.61%
Online consultations	161	26.39%
Total	610	100%

### 3.3. Hospital Appointment Booking Services

**Figure 2:**
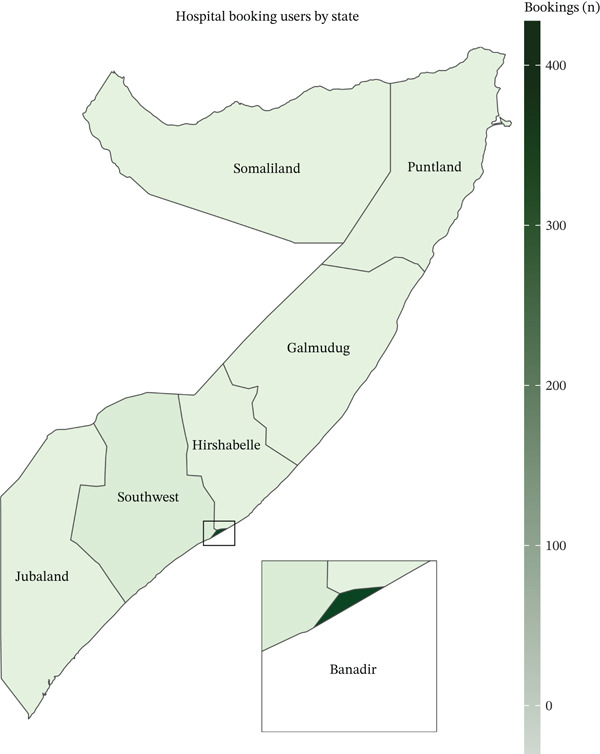
Regional analysis of hospital booking service utilization in Somalia.

Within the hospital booking service user subset (*n* = 449), the age distribution was consistent with overall user demographics, with 65.03% (*n* = 292) aged 1–30 years (Table 3). Females comprised a slight majority (51.89%, *n* = 233). Dental services emerged as the most frequently requested specialty, comprising 42.98% (*n* = 193) of bookings, followed by orthopedic surgery (10.24%, *n* = 46) and ophthalmology (9.58%, *n* = 43). The geographical distribution of booking services showed an even stronger concentration in the Banadir region (Figure 2), at 94.88% (*n* = 426).

**Table 3:** Hospital appointment booking services through digital platform (n = 449).

Variable	Frequency	Percentage
Age (years)		
1–30	292	65.03%
31–45	72	16.04%
46+	85	18.93%
Mean ± SD	31.04 ± 18.85	
Sex		
Male	216	48.11%
Female	233	51.89%
State		
Banadir	426	94.88%
Southwest	19	4.23%
Hirshabelle	2	0.45%
Jubaland	1	0.22%
Puntland	1	0.22%
Booked specialist		
Dentist	193	42.98%
Orthopedic surgeon	46	10.24%
Ophthalmologist	43	9.58%
Pediatrician	39	8.69%
OBS/GYN doctor	31	6.9%
Internal medicine doctor	20	4.45%
ENT surgeon	18	4.01%
Urologist	17	3.79%
General surgeon	15	3.34%
Neurologist	11	2.45%
Gastroenterologist	7	1.56%
Internal medicine	5	1.11%
Dermatologist	2	0.45%
Cardiologist	2	0.45%

### 3.4. Remote Medical Consultation Characteristics

**Figure 3:**
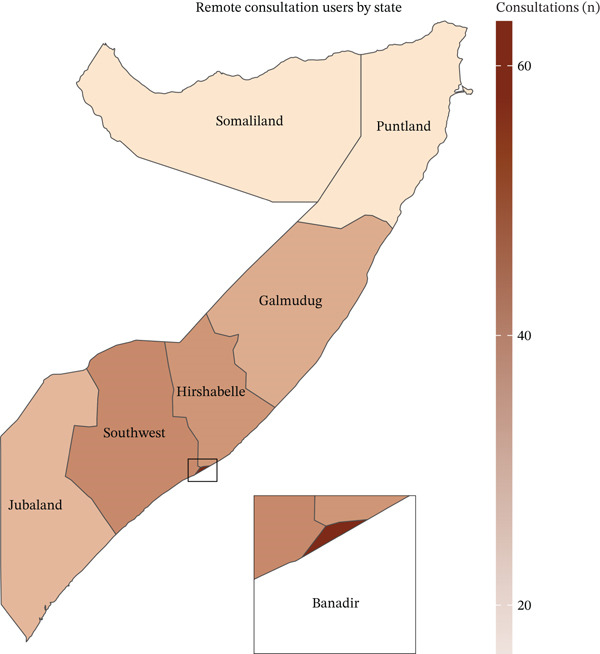
Distribution of remote medical consultations by state.

Remote consultation services (*n* = 161) demonstrated distinct utilization patterns (Table 4). The age distribution was 59.01% (*n* = 95) of the users aged 1–30 years, with a mean age of 30.45 years (SD = 16.53). Male users predominated this service category at 58.39% (*n* = 94). Geographical analysis showed that the Banadir state represented the highest proportion at 40.37% (*n* = 65), followed by the Southwest state at 19.25% (*n* = 31), Hirshabelle at 16.77% (*n* = 27), Galmudug at 12.42% (*n* = 20), and Jubaland at 9.94% (*n* = 16), whereas both Somaliland and Puntland represented 0.62% (*n* = 1) of consultations (Figure 3). Consultations regarding infections constituted the largest proportion (36.0%, *n* = 58), followed by orthopedics and physiotherapy (17.4%, *n* = 28) and obstetrics and gynecology (14.3%, *n* = 23).

**Table 4:** Distribution and characteristics of remote medical consultations (n = 161).

Variable	Frequency	Percentage
Age (years)		
1–30	95	59.01%
31–45	36	22.36%
46+	30	18.63%
Mean ± SD	30.45 ± 16.53	
Sex		
Male	94	58.39%
Female	67	41.61%
State		
Banadir	65	40.37%
Southwest	31	19.25%
Hirshabelle	27	16.77%
Galmudug	20	12.42%
Jubaland	16	9.94%
Somaliland	1	0.62%
Puntland	1	0.62%
Department/specialty		
Infections	58	36.0%
Orthopedics and physiotherapy	28	17.4%
Obstetrics and gynecology	23	14.3%
Internal medicine	21	13.0%
Dermatology	11	6.8%
Neurology and psychiatry	11	6.8%
Special senses (ENT issues, eye issues, and dental issues)	9	5.7%

### 3.5. IVR Self‐Management Service Usage

**Figure 4:**
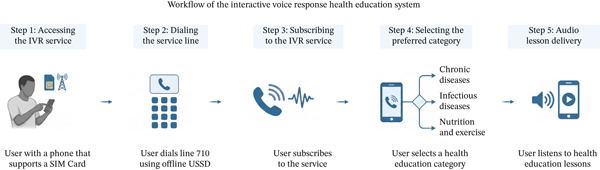
Workflow of the interactive voice response health education system. *Source:* Created in BioRender. Ahmed, M. M. (2026) https://BioRender.com/c3sz2qt.

The IVR self‐management service demonstrated substantial usage with 157,660 total interactions (Figure 4; Table 5). Chronic condition management dominated service usage, accounting for 47% (*n* = 74,436) of all interactions, with diabetes management being the most frequently accessed topic at 23% (*n* = 36,916). Lifestyle‐related content, specifically food and exercise, represented 27% (*n* = 42,760) of the total usage. Infectious disease information accounted for 26% (*n* = 40,464) of the interactions, with malaria information being the most accessed at 10% (*n* = 15,512).

**Table 5:** IVR self‐management service usage (n = 157,660).

Category	Subcategory	Frequency	Percentage
Chronic	Cholesterol	7832	5%
	Hypertension	14,620	9%
	Liver	15,068	10%
	Diabetes	36,916	23%
Total (chronic)		74,436	47%
Lifestyle	Food and exercise	42,760	27%
Infectious	Cholera	8736	6%
	Hepatitis	8612	5%
	HIV/AIDS	7604	5%
	Malaria	15,512	10%
Total (infectious)		40,464	26%
Overall total		157,660	100%

## 4. Discussion

This study examined the implementation of Baano Healthcare Technology′s integrated telemedicine platform in Somalia and revealed several patterns and implications for healthcare delivery in resource‐limited settings. The findings demonstrate predominant youth engagement in telemedicine services, with 63.44% of users aged 1–30 years, reflecting Somalia′s demographic structure, where 75% of the population consists of young individuals aged less than 30 years [20]. This youth predominance aligns with the broader digital transformation in Somalia, where increasing mobile phone penetration and internet accessibility have created favorable conditions for digital health solutions [16]. The equal gender distribution in service utilization, with 50.82% males and 49.18% females, suggests that telemedicine platforms can provide equitable healthcare access across gender lines in Somalia′s cultural context. This balanced distribution is particularly noteworthy given the traditional barriers to accessing healthcare for women in many parts of Somalia [21]. A notable geographical distribution pattern emerged in service utilization, with the Banadir region accounting for 80.49% of all users.

This urban predominance, particularly in hospital booking services, where Banadir represented 94.88% of appointments, reflects the concentration of healthcare facilities and strong telecommunication infrastructure in the capital region [22, 23]. Somalia shows stark geographic and socioeconomic gradients. The 2022–2023 Harmonized Health Facility Assessment recorded state‐level facility densities as low as 0.58 per 10,000 residents in Galmudug and 0.33 per 10,000 in Banadir, versus 0.90 per 10,000 in Hirshabelle [11]. Despite its low per‐capita ratio, Banadir concentrates 45 of the country′s 155 hospitals (≈29%), including 6 of the 8 national referral hospitals and 34 of the 57 specialty hospitals, illustrating a heavy urban clustering of higher level care [11, 14]. Poverty mirrors this pattern: Analysis of the 2022 Somali Integrated Household Budget Survey shows 78% of nomadic households live below the national poverty line, compared with 46% of urban residents in Banadir [24].

Connectivity gaps compound the divide; DataReportal′s Digital 2023 puts overall internet use at just 9.8% of the population [25], and GSMA′s State of Mobile Internet Connectivity 2023 finds that, across low‐ and middle‐income countries, adults in rural areas are 29% less likely than their urban peers to use mobile internet [26]. Taken together, these shortfalls in care availability, household income, and digital access help explain why Baano bookings and video consultations cluster in Banadir and Somaliland, whereas IVR interactions, which require only basic voice or USSD coverage, are more evenly distributed across low‐connectivity states. However, this urban‐centric utilization also highlights the persistent disparities in healthcare access between urban and rural areas in Somalia, similar to the challenges observed in other fragile contexts, such as Yemen, where only 26.7% of the population has internet access and significant urban–rural digital divides exist [27].

The analysis of service preferences revealed that users predominantly opted for hospital booking services (73.61%) over direct online consultations (26.39%). Dental services made up 42.98% of all appointments on the platform, not because infectious problems are a low priority for Somalis, but because most operative oral health procedures still require a chair‐side visit, whereas many infectious symptoms can be assessed and triaged through video calls. A recent study on Somali oral health workforce reported that “virtually all licensed dentists are clustered in Mogadishu/Banadir, leaving the rest of the country with fewer than one dentist per 100,000 inhabitants and no public dental units” [28]. In this context, Baano′s booking engine provides one of the few practical pathways for patients outside the capital to reach scarce dental specialists, which explains the disproportionately high demand for dental slots. In contrast, infections remain the single most common reason for video consultations (36%), confirming ongoing concern; their lower share of hospital bookings reflects users′ deliberate channel choice rather than diminished perceived importance. Although this digital transformation in healthcare booking shows promise, it highlights an important gap in the current system. There is a critical need to establish validation and regulatory mechanisms for healthcare providers, particularly dental specialists, who are listed on booking platforms [29]. Such regulations would ensure that only verified and qualified practitioners are accessible through the system, thereby protecting patient safety and maintaining service quality standards [30, 31].

Remote consultations demonstrated a broader geographical reach than hospital bookings, with significant utilization across multiple regions, including the Southwest state (19.25%) and Hirshabelle (16.77%). This distribution pattern suggests that telemedicine can effectively bridge the geographical healthcare access gaps when infrastructure permits. The predominance of infection consultations (36.0%), followed by orthopedics and physiotherapy (17.4%), reflects both the high burden of infectious diseases in Somalia and the prevalence of musculoskeletal conditions in the predominantly young population [32, 33]. The significant proportion of orthopedic and physiotherapy consultations aligns with the demographic profile, where 63.44% of users are aged 1–30 years, suggesting a correlation with sports‐related injuries and physical activities common in this age group. Substantial engagement with IVR self‐management services, totaling 157,660 interactions, revealed important patterns in health information‐seeking behaviors. The high interest in chronic condition management, particularly diabetes (23%) [34], reflects the growing burden of noncommunicable diseases in Somalia [35, 36].

The significant utilization of lifestyle‐related content (27%) suggests an increasing awareness of preventive health measures and wellness management among the Somali population [37]. These findings demonstrate the potential and challenges of implementing telemedicine services in resource‐limited settings such as Somalia. Although the platform shows promise in expanding healthcare access, particularly in urban areas, significant work remains to ensure the equitable distribution of these benefits across all population segments and geographical regions. Success in urban areas provides a model for expanding digital health services to underserved regions, although this requires addressing infrastructure limitations, regulatory frameworks, and cultural barriers to adoption [30]. In the Somali context, equitable rural expansion will likely require a phased strategy that goes beyond internet infrastructure alone. Such a strategy should include strengthening low‐bandwidth service channels, such as IVR and USSD, integrating telemedicine with existing primary healthcare facilities, engaging community health workers and local referral networks, and developing subsidized or community‐supported access models for poorer and nomadic populations [30, 38]. Partnerships with mobile network operators and local health authorities may also be important for extending reach to hard‐to‐access districts, where smartphone‐based care remains unrealistic in the near term [30, 38].

The notably low utilization among older populations (18.85% aged 46 years and above) highlights the critical need for targeted digital literacy programs and user‐friendly interfaces tailored to elderly users [39, 40]. Furthermore, the absence of comprehensive telemedicine policies and regulations in Somalia necessitates the development of standardized guidelines for digital healthcare delivery, including aspects of data protection, service quality standards, and professional accountability [30]. These findings suggest that the strategic implementation of telemedicine services, coupled with appropriate policy frameworks and digital education initiatives, could significantly contribute to increasing Universal Health Coverage (UHC) in Somalia by improving healthcare accessibility, particularly for underserved populations who currently face geographical and infrastructural barriers to traditional healthcare services [41]. Additionally, the integration of emerging technologies, such as digital twins, could enhance the optimization of healthcare delivery systems in Africa, enabling better system monitoring and resource allocation in resource‐limited settings [42]. From a longer‐term perspective, the impact of telemedicine in Somalia should not only be judged by early utilization volumes but also by whether these platforms become embedded within referral pathways, improve continuity of care, support chronic disease follow‐up, and reduce avoidable geographic inequities over time. Therefore, sustained impact will depend on regulatory stewardship, financial sustainability, interoperability with broader health systems, and deliberate monitoring of who is reached and who continues to be left behind [38, 43].

This study has several limitations. First, the 4‐month observation period and cohort of 610 users of video consultations and hospital bookings may not capture seasonal variations, thereby limiting statistical power. Second, the dataset was predominantly skewed towards Banadir, which introduced the risk of selection bias and restricted the generalizability of the findings to a national context. Third, the IVR channel lacks important data, such as subscriber age, gender, and unique identifiers, resulting in unstratified channel comparisons and hindering the identification of repeat callers. Fourth, the registration process did not collect socioeconomic or clinical data (e.g., income, occupation, and comorbidity), thus precluding equity analyses along these dimensions and potentially obscuring access barriers. Fifth, the study did not include qualitative feedback from users and providers, which could clarify the contextual factors influencing uptake and better explain adoption barriers, service preferences, and implementation experiences across different user groups and care settings. Sixth, as external researchers, we were granted access only to aggregated operational data for the digital consultation and hospital booking services, and individual‐level anonymised records were not made available by the platform provider. For the IVR component, access to detailed interaction logs was also limited because the service operates through USSD infrastructure managed by the telecommunications provider; therefore, raw timestamp‐level logs were unavailable to the study team. Finally, this study did not assess cost‐effectiveness, leaving questions of economic sustainability and scalability unresolved.

Future research should extend the follow‐up period, broaden geographic sampling, incorporate qualitative methods, gather comprehensive demographic and clinical data, and conduct economic evaluations to provide a more detailed evidence. Qualitative inquiry involving both users and providers would be valuable for understanding perceived barriers, facilitators, and preferences that are not captured in routine operational datasets. Future studies would also benefit from broader access to anonymised individual‐level records and IVR log‐level data, where feasible and permitted, to enhance transparency and enable more granular analyses. Despite these limitations, the findings provide valuable insights into future digital health initiatives in resource‐limited settings, highlighting both the opportunities and challenges of leveraging technology to improve healthcare access and outcomes. Future research should focus on long‐term evaluation, broader geographical coverage, and comprehensive assessment of clinical outcomes and cost‐effectiveness to better inform policies and implementation strategies.

## 5. Conclusion

The implementation of integrated telemedicine services in Somalia has yielded promising outcomes in urban healthcare delivery, particularly through the Baano Healthcare Technology platform. Balanced demographic engagement, especially among the youth, demonstrates successful digital health adoption. Urban centers, notably the Banadir region, showed robust utilization patterns across hospital bookings and consultations, although this highlighted the need for expanded rural coverage. Urban concentration reflects not only provider availability and digital access but also deeper socioeconomic divides, underscoring the need for inclusive digital strategies. The predominance of dental service bookings points to both user needs and supply side limitations, emphasizing the necessity of systematic provider validation to ensure care quality and trust. Remote consultations have proven effective in bridging geographical healthcare gaps, particularly in managing infections and orthopedic cases. Extensive engagement with IVR self‐management services, especially for chronic condition management, indicates growing health awareness and self‐care practices. However, their limited use among older adults suggests the need for targeted digital literacy initiatives and age‐friendly platform designs. Moving forward, key priorities include addressing infrastructure limitations in rural areas, developing comprehensive regulatory frameworks, and ensuring equitable service distribution across regions. Practical strategies for rural scale‐up should include strengthening low‐connectivity service models, linking telemedicine with primary care and community‐based delivery platforms, and creating targeted support mechanisms for populations facing financial, geographic, and digital barriers. Additionally, collecting richer user data, including socioeconomic and clinical characteristics, is essential for monitoring equity and impact. Although the platform′s success in urban areas provides a scalable model, realizing its full potential will require sustained investment, multisectoral partnerships, and inclusive health policies to extend digital health benefits to Somalia′s most underserved populations. In the long term, the value of integrated telemedicine in Somalia will depend on whether it evolves from an urban‐centered innovation into an equitable and sustainable component of the national health system.

## Author Contributions

N.I.D. and M.M.A. conceptualized this idea. The study design was developed collaboratively by N.I.D. and M.M.A. Material preparation and data collection were performed by N.I.D. M.M.A. analyzed and interpreted the data. The initial draft was composed by M.M.A. A.A.M. contributed to the reviewing and editing of the subsequent versions of the manuscript.

## Funding

No funding was received for this manuscript.

## Ethics Statement

Ethical approval for this study was obtained from the Institutional Review Board (IRB) of SIMAD University, Mogadishu, Somalia (Reference: 2024/SU‐IRB/FMHS/P065, dated October 30, 2024). As this study involved the analysis of de‐identified operational data, individual patient consent was waived by the IRB.

## Consent

Consent for publication was obtained from Baano Healthcare Technology for the use of the operational data in this study.

## Conflicts of Interest

The authors declare no conflicts of interest.

## Data Availability

The data that support the findings of this study are available from Baano Healthcare Technology. Restrictions apply to the availability of these data, which were used under license for this study. Data are available from the authors with the permission of Baano Healthcare Technology.
